# Celastrol directly binds with VAMP7 and RAB7 to inhibit autophagy and induce apoptosis in preadipocytes

**DOI:** 10.3389/fphar.2023.1094584

**Published:** 2023-03-07

**Authors:** Chenshu Liu, Na Li, Meixiu Peng, Kan Huang, Dongxiao Fan, Zhengde Zhao, Xiuyi Huang, Yunchong Liu, Sifan Chen, Zilun Li

**Affiliations:** ^1^ Division of Vascular Surgery, The First Affiliated Hospital of Sun Yat-Sen University, Guangzhou, Guangdong, China; ^2^ National-Guangdong Joint Engineering Laboratory for Diagnosis and Treatment of Vascular Diseases, The First Affiliated Hospital of Sun Yat-Sen University, Guangzhou, Guangdong, China; ^3^ Guangdong Provincial Key Laboratory of Malignant Tumor Epigenetics and Gene Regulation, Guangdong-Hong Kong Joint Laboratory for RNA Medicine, Medical Research Center, Sun Yat-Sen Memorial Hospital, Sun Yat-Sen University, Guangzhou, China; ^4^ Nanhai Translational Innovation Center of Precision Immunology, Sun Yat-Sen Memorial Hospital, Foshan, China

**Keywords:** celastrol, VAMP7, Rab7, apoptosis, autophagy, adipocyte, obesity

## Abstract

Obesity is one of the most prevalent chronic metabolic diseases, and induction of apoptosis in preadipocytes and adipocytes is a potential strategy to treat obesity. Celastrol represents one of the most robust anti-obesity phytochemicals so far, yet its direct binding target remains elusive. Here, we determined that celastrol could induce apoptosis in preadipocytes *via* mitochondrial mediated pathway. Further study clarified that celastrol inhibited the fusion of autophagosome and lysosome to prohibit autophagy, leading to cell apoptosis. By conducting virtual screening and genetic manipulation, we verified that overexpression of VAMP7 and RAB7 could block the effects of celastrol on inhibiting autophagy and inducing apoptosis. The Surface Plasmon Resonance study confirmed the direct binding of celastrol with VAMP7 and RAB7. The functional study illustrated the inhibition of RAB7 GTPase activity after celastrol treatment. Moreover, celastrol induced comparable apoptosis in murine epididymal adipose tissue, human preadipocytes and adipocytes, but not in human hepatocytes. An inhibitory effect on differentiation of human primary visceral preadipocytes was also observed. In conclusion, celastrol exhibited inhibitory effect of autophagy *via* direct binding with VAMP7 and RAB7, leading to an increase in preadipocytes apoptosis. These results advance our understanding in the potential application of celastrol in treating obesity.

## 1 Introduction

Obesity is one of the most prevalent chronic diseases among the world, and threatens human health with tremendous social-economics cost (Ng et al., 2014). More than 1.9 billion adults are overweight, and over 650 million are obese worldwide (https://www.who.int/news-room/fact-sheets/detail/obesity-and-overweight). In obesity, adipose tissue exhibits hypertrophy and pathological adipogenesis, which lead to insulin resistance and chronic inflammation (Piche et al., 2020). In obesity, the proliferation and differentiation of preadipocytes to adipocytes are overrepresented, while the mature adipocytes enlarge their volume to store the increased triacylglycerols (Ali et al., 2013). Hence, decreasing fat mass with activation of lipolysis, inhibition of adipogenesis or apoptosis of preadipocytes and adipocytes, are considered as potential strategies to treat obesity. In perspective of apoptosis in preadipocytes and adipocytes, several phytochemicals were proven to induce apoptosis of preadipocytes, which decreased the fat mass accumulation (Hsu and Yen, 2006; Yang et al., 2007; Chen et al., 2012; Zhang and Huang, 2012; Lone and Yun, 2017; Wu et al., 2019), suggesting them as promising compounds in treating obesity.

Celastrol is a natural friedelane pentacyclic triterpenoid, which can be extracted from some celastraceae plants such as *Tripterygium wilfordii* and *Celastrus orbiculatus.* (Xu et al., 2021). It is also one of the most robust anti-obesity phytochemicals that has been reported so far, yet its direct target in this regard remains unknown. Liu et al. had reported that up to 45% weight loss was observed in obese mice treated with celastrol (Liu et al., 2015), which is even more potent than 35%–40% weight loss in mice after bariatric surgery (Liou et al., 2013; Mokadem et al., 2014; Ryan et al., 2014). Despite the strong anti-obesity effect of celastrol, identification of its direct target remains challenging. To date, only adenylyl cyclase-associated protein 1 (Zhu et al., 2021) and nuclear receptor subfamily four group A member 1 (Hu et al., 2017) were reported to be able to directly bind with celastrol, yet neither was verified as the direct target of its anti-obesity effect. Hence, identification of the target would significantly advance its mechanistic investigation and clinical translation.

Autophagy plays a pivotal role in preadipocytes differentiation and fat accumulation. Autophagy is an essential mechanism for cells to maintain physiological homeostasis, including turnover of the protein and nutrients, and elimination of the potential hazards (Doherty and Baehrecke, 2018). Studies showed that damage of autophagic flux would inhibit preadipocytes differentiation and subsequently induce apoptosis, indicating a vital role of autophagy in adipogenesis (Baerga et al., 2009; Singh et al., 2009; Zhang et al., 2009).

In our study, we demonstrated that celastrol could induce apoptosis of preadipocytes and mature adipocytes. With autophagic flux assay, gene manipulation and small molecule-protein binding assay, we found that celastrol could directly bind with vesicular transport related proteins, namely, VAMP7 and RAB7, inhibit the fusion of autophagosome and lysosome, leading to impaired autophagic flux and subsequent induction of cell apoptosis in preadipocytes. This study determined a direct effect of celastrol on preadipocytes and uncovered its direct binding target, advancing the potential application of celastrol in treating obesity.

## 2 Materials and methods

### 2.1 Cell culture

The cell lines of murine 3T3-L1 preadipocytes and human hepatocytes HL-7702 presented in this study were obtained from the National Collection of Authenticated Cell Cultures of China (Shanghai, China). The studies involving human primary visceral preadipocytes and adipocytes of human participants were reviewed and approved by the Institutional Review Board of The First Affiliated Hospital of Sun Yat-sen University (Guangzhou, China). The patients/participants provided their written informed consent to participate in this study. Cells were cultured in normal cell culture incubator. Murine 3T3-L1 preadipocytes and human hepatocytes HL-7702 were cultured in Dulbecco’s modified Eagle’s medium (DMEM, Gibco, United States) supplemented with 10% fetal bovine serum (FBS, Gibco, United States), 100 U/mL of penicillin and 100 μg/mL of streptomycin. The human primary visceral preadipocytes and adipocytes were cultured in DMEM/F12 medium without phenol red (Gibco, United States), supplemented with 10% FBS, 100 U/mL of penicillin and 100 μg/mL of streptomycin. For adipocytes differentiation, the classic protocol of preadipocytes differentiation was followed (He et al., 2021). In brief, 3T3-L1 preadipocytes were cultured 2 days in DMEM medium (0.5 mmol/L IBMX, 1 μmol/L dexamethasone, 10 μg/mL insulin, and 10% FBS), and then 2 days in DMEM medium (5 μg/mL insulin and 10% FBS), once reached full confluence. Afterwards, the cells were maintained in DMEM medium (10% FBS) for 4 days till full differentiation. DMEM/F12 medium without phenol red was applied for human visceral preadipocytes in this differentiation protocol.

### 2.2 Animals

Four-week-old male C57BL/6 mice were housed under normal specific pathogen free (SPF) conditions with unrestricted access to food and water and were fed with 60% high fat diet for 24 weeks to induce diet-induced obesity. After induction, mice were randomly divided into five groups, 1) chow diet + vehicle, 2) high fat diet + vehicle, 3) high fat diet + celastrol, 4) pair-feeding + vehicle and 5) pair-feeding + celastrol (*n* = 4). For 2 weeks intervention of celastrol (100 μg/kg/day, i. p.), mice in two pair-feeding groups only received equal amount diet comparing with high fat diet + celastrol treatment group. After 6 h fasting, all mice were sacrificed with anesthesia to harvest their epididymal adipose tissue. All animal studies were approved by the Institutional Animal Care and Use Committees of the First Affiliated Hospital of Sun Yat-sen University.

### 2.3 Cell treatment

For RFP-GFP-LC3 adeno-associated virus (Hanbio, China) infection, 3T3-L1 preadipocytes in 12-well plate were transfected according to the manufacturers’ instructions, celastrol were given 48 h after transfection, and harvested after 24 h for the following experiments. For siRNA and plasmids transfection, Lipofectamine 2000 (Invitrogen, United States) was applied according to the manufacturers’ instructions. Preadipocytes in 12-well plate were transfected with each RAB7, VAMP7 or VTI1B plasmids for 48 h for overexpression, and then subjected to celastrol for additional 24 h. Ten nM of the RAB5C siRNA was applied for each well in 12-well plate, added together with celastrol, and subjected to the following experiments after 24 h. All control wells received the corresponding blank plasmids or scramble siRNA.

### 2.4 Apoptosis assay

Flow cytometry was applied for cell apoptosis assay. In brief, preadipocytes after treatment were harvested, washed twice with cold PBS and resuspended with buffer to reach 1 × 10^5^ cells/mL. Suspension was further incubated with dyes from Annexin V, FITC Apoptosis Detection Kit (Dojindo, China). The percentages of distribution of normal (Annexin V−/PI−), apoptotic (Annexin V+/PI− and Annexin V +/PI+) and necrotic cells (Annexin V−/PI+) were calculated.

### 2.5 Mitochondrial membrane potential assay

Mitochondrial membrane potential assay was applied using a JC-10 based commercial kit (Biosharp, China). JC-10 exhibits potential-dependent aggregate status in normal mitochondria membrane (red), and monomer status in abnormal mitochondria membrane (green). 3T3-L1 preadipocytes in 12-well plate were administrated with celastrol for 24 h and then subjected to JC-10 dye loading solution at 37°C in a 5% CO_2_ incubator for 20 min, avoid light. The plates were further observed with fluorescence microscope (Leica DMi8, United States).

### 2.6 Terminal deoxynucleotidyl transferase dUTP nick end-labelling (TUNEL) staining

For *in vitro* TUNEL assay, preadipocytes were treated with celastrol for 24 h and dyed by One Step TUNEL Apoptosis Assay kit (Beyotime Institute of Biotechnology, China) (Liu et al., 2019). Preadipocytes were fixed with 4% paraformaldehyde at room temperature for 30 min. Untreated cells were pre-incubated with DNase I recombinant (5 μg/mL) for 10 min at room temperature to serve as a positive control. Preadipocytes were further incubated with TUNEL reaction mixture for 60 min at 37°C in dark. The TUNEL-positive nuclei (green) was observed under a fluorescence microscope (Leica DMI8, United States).

For *in vivo* TUNEL assay, *In situ* Cell Death Detection Kit (Roche, Switzerland) was applied for epididymal adipose tissue. The histological sections were incubated with TUNEL reaction mixture for 60 min at 37°C in the dark, incubated with Converter-POD antibody (1:500) for 30 min at 37°C, followed by DAB substrate incubation for 10 min at room temperature, and then mounted with PBS/glycerol. The number of TUNEL-positive nuclei (brown) was calculated from six random fields of each sections under a light microscope (Zeiss Axio Observer Z1, German).

### 2.7 LysoTracker red staining

Preadipocytes were treated with celastrol, 40 μM chloroquine or 200 nM bafilomycin A1 for 24 h and stained with LysoTracker Red fluorescence probes (Solarbio, China). After compounds treatment, cells were incubated with DMEM complete media containing LysoTracker Red dye (25 nM) and Hoechst 33258 Staining Dye for 20 min at 37°C in dark. Cells were further observed under a fluorescence microscope (Leica DMI8, United States).

### 2.8 Immunofluorescence staining

Preadipocytes infected with RFP-GFP-LC3 adeno-associated virus were treated with 1 μM celastrol, 40 μM chloroquine or 200 nM bafilomycin A1 for 24 h. Cells were fixed with 4% paraformaldehyde and blocked with 5% BSA, then subjected to primary antibody LAMP1 (Cell Signaling Technology, United States, Cat #9091, RRID:AB_2687579, 1:100 dilution) overnight, followed by incubation with Alexa Fluor 647-conjugated goat anti-rabbit IgG antibody (Abcam, United States, Cat # ab150079, RRID:AB_2722623, 1:500 dilution). Finally, the autophagosomes were observed under a fluorescence microscope (Leica DMI8, United States). Yellow against red puncta ratio was determined by the exact puncta numbers in five random fields of one slice obtained from YFP and RHOD channels separately. The Pearson correlation coefficient (PCC) for the colocalization of RFP-LC3 and Alexa Fluor 647-LAMP1 was calculated with the Coloc2 module of ImageJ (National Institutes of Health, United States, RRID:SCR_003070) (Elimam et al., 2019).

### 2.9 Electron microscopy

3T3-L1 preadipocytes were treated with 1 and 2 μM celastrol or 40 μM chloroquine for 12 h and were fixed with 2.5% glutaraldehyde in sodium phosphate buffer (pH 7.4) for 30 min at room temperature. The samples were then dehydrated in a series of aqueous alcohol solutions, and finally 100% alcohol and embedded in epoxy resin. Ultrathin sections cut in a Leica ultramicrotome (Leica UC7, United States) were stained with lead citrate and uranyl acetate and observed using a HT7800 electron microscope (HITACHI, Japan).

### 2.10 Oil Red O staining

Oil Red O staining was performed as described (Huang et al., 2021). During differentiation, 3T3-L1 preadipocytes were treated with 200 nM–1,000 nM celastrol constantly. After full differentiation, cells were stained with Oil red O dye (Sigma, United States) at room temperature for 15 min, followed by de-staining with 60% isopropyl alcohol for 5 s. Oil red O staining was obtained with a light microscope, and the statistics was calculated by ImageJ software (National Institutes of Health, United States, RRID:SCR_003070).

### 2.11 Real-time quantitative PCR (RT-qPCR)

The 3T3-L1 preadipocytes were treated with 2 and 4 μM celastrol for 16 h, then subjected to AG RNAex Pro Reagent (Accurate Biotechnology, China) for total RNA extraction and further reverse transcription. RT-PCR was performed using SYBR staining (Accurate Biotechnology, China) in a LightCycle480 II thermal cycler (Roche, Switzerland). Relative gene expression was normalized against *Actin,* with control group value set to 1. The primer sequences are *Bax*-forward primer: AGG​ATG​CGT​CCA​CCA​AGA​AGC​T, -reverse primer: TCC​GTG​TCC​ACG​TCA​GCA​ATC​A; *Bcl2*-forward primer: CCT​GTG​GAT​GAC​TGA​GTA​CCT​G, -reverse primer: AGC​CAG​GAG​AAA​TCA​AAC​AGA​GG; *Chop*-forward primer: GGA​GGT​CCT​GTC​CTC​AGA​TGA​A, -reverse primer: GCT​CCT​CTG​TCA​GCC​AAG​CTA​G; *P62*-forward primer: GCT​CTT​CGG​AAG​TCA​GCA​AAC​C, -reverse primer: GCA​GTT​TCC​CGA​CTC​CAT​CTG​T; *Lc3b*-forward primer: GTC​CTG​GAC​AAG​ACC​AAG​TTC​C, -reverse primer: CCA​TTC​ACC​AGG​AGG​AAG​AAG​G; *Becn1*-forward primer: CAG​CCT​CTG​AAA​CTG​GAC​ACG​A, -reverse primer: CTC​TCC​TGA​GTT​AGC​CTC​TTC​C; *Hif1a*-forward primer: CCT​GCA​CTG​AAT​CAA​GAG​GTT​GC, -reverse primer: CCA​TCA​GAA​GGA​CTT​GCT​GGC​T; *Hif2a*-forward primer: GGA​CAG​CAA​GAC​TTT​CCT​GAG​C, -reverse primer: GGT​AGA​ACT​CAT​AGG​CAG​AGC​G; *Bnip3*-forward primer: GCT​CCA​AGA​GTT​CTC​ACT​GTG​AC, -reverse primer: GTT​TTT​CTC​GCC​AAA​GCT​GTG​GC; *Vim*-forward primer: CGG​AAA​GTG​GAA​TCC​TTG​CAG​G, -reverse primer: AGC​AGT​GAG​GTC​AGG​CTT​GGA​A; *Col3a*-forward primer: GAC​CAA​AAG​GTG​ATG​CTG​GAC​AG, -reverse primer: CAA​GAC​CTC​GTG​CTC​CAG​TTA​G; *Actin*-forward primer: CAT​TGC​TGA​CAG​GAT​GCA​GAA​GG, -reverse primer: TGC​TGG​AAG​GTG​GAC​AGT​GAG​G.

### 2.12 Western blotting

For whole cell protein extraction, preadipocytes and fat tissue were lysed in RIPA buffer (50 mM Tris-HCl, pH 7.4, 150 mM NaCl, 1% NP-40, 0.1% SDS) with protease inhibitors and phosphatase inhibitors. For cytoplasm protein extraction, cells were prepared in RSB buffer (10 mmol/L Tris (pH 7.4), 10 mmol/L NaCl, 3 mmol/L MgCl2, 0.5% NP40) with protease inhibitors and phosphatase inhibitors. The bicinchoninic acid (BCA) (ComWin Biotech, China) was used to measured protein concentration. For RAB7 GTPase activity assay, 3T3-L1 preadipocytes were treated using Rab7 Pull-Down Activation Assay Kit (NewEast Biosciences, United States). After 1 and 2 μM celastrol treatment for 16 h, whole cell proteins from preadipocytes were harvested using lysis buffer from the kit, and a half extracts from the control group was incubated with GDP for 30 min at 30°C to serve as negative control. The extracts were further incubated with protein A/G Agarose beads conjugating anti-Rab7-GTP antibody for 1 h at 4°C. The beads were washed and resuspended with SDS-PAGE loading buffer. Equal amounts of protein were subjected to SDS-PAGE and transferred to a PVDF membrane, then the membrane was incubated in 5% milk in Tris-buffered saline for 1 h at room temperature, followed by primary antibodies of Cytochrome C (Cell Signaling Technology, United States, Cat # 11940, RRID:AB_2637071, 1:1,000 dilution), cleaved-Caspase3 (Cell Signaling Technology, United States, Cat #9664, RRID:AB_2070042, 1:1,000 dilution), P62 (Sigma, United States, Cat #P0067, RRID:AB_1841064, 1:1,000 dilution), LC3 I/II (Sigma, United States, Cat #L7543, RRID:AB_796155, 1:1,000 dilution), LAMP1 (Cell Signaling Technology, United States, Cat #9091, RRID:AB_2687579, 1:1,000 dilution), RAB7 (Cell Signaling Technology, United States, Cat #9367, RRID:AB_1904103, 1:1,000 dilution), *β*-actin (Cell Signaling Technology, United States, Cat #4970, RRID:AB_2223172, 1:1,000 dilution), GAPDH (Cell Signaling Technology, United States, Cat #5174, RRID:AB_10622025, 1:1,000 dilution) and Flag (Cell Signaling Technology, United States, Cat #14793, RRID:AB_2572291, 1:1,000 dilution) overnight at 4°C, and a secondary antibody (1:5,000 dilution) conjugated with horseradish peroxidase (Cell Signaling Technology, United States, Cat #7074, RRID:AB_2099233) for 1 h at room temperature. Membranes were developed with chemiluminescent ECL reagents (Millipore, United States). The relative expression of target protein to the control was determined by ImageJ software (National Institutes of Health, United States, RRID:SCR_003070).

### 2.13 Virtual docking

The crystal structure of the apo form of human VAMP7 (PDB: 2VX8), RAB5C (PDB: 1Z0D), RAB7 (PDB: 1VG1), VTI1B (PDB: 2V8D), VAMP8 (PDB: 3ZYM), SNP29 (PDB: 4WY4), PLEKHM1 (PDB: 5DPT) and SNARE-complex (PDB: 3RK2) were applied for molecular docking. The AutoDockTools-1.5.7 was used for virtual docking of abovementioned proteins and celastrol (Rogério et al., 2022).

### 2.14 Surface Plasmon Resonance (SPR)

The SPR assays were performed to analyze the interactions between the compounds and VAMP7, RAB7 proteins (Sino Biological, China) by using a Biacore T100 machine with Sensor Chip CM5 (GE Healthcare, United States) at 25°C. Two proteins were immobilized onto CM5 chips, and sensorgrams were recorded by injecting various concentrations of compounds. The binding kinetics (Kd) was analyzed with the software BIA evaluation Version 4.1 (GE Healthcare, United States).

### 2.15 Statistical analysis

All data were shown as mean ± SEM. All results shown were representative of at least three independent biological replicates of experiments. Data were analyzed with SPSS version 24.0 software (IBM Corp, United States, RRID:SCR_002865). One-way analysis of variance (ANOVA) was performed for the comparison of multiple groups. Bonferroni post-hoc testing was used following ANOVA for analyzing all pairwise comparisons between groups. *p* < 0.05 was considered a statistically significant difference.

## 3 Results

### 3.1 Celastrol induced 3T3-L1 preadipocytes apoptosis *via* mitochondrial mediated pathway

**FIGURE 1:**
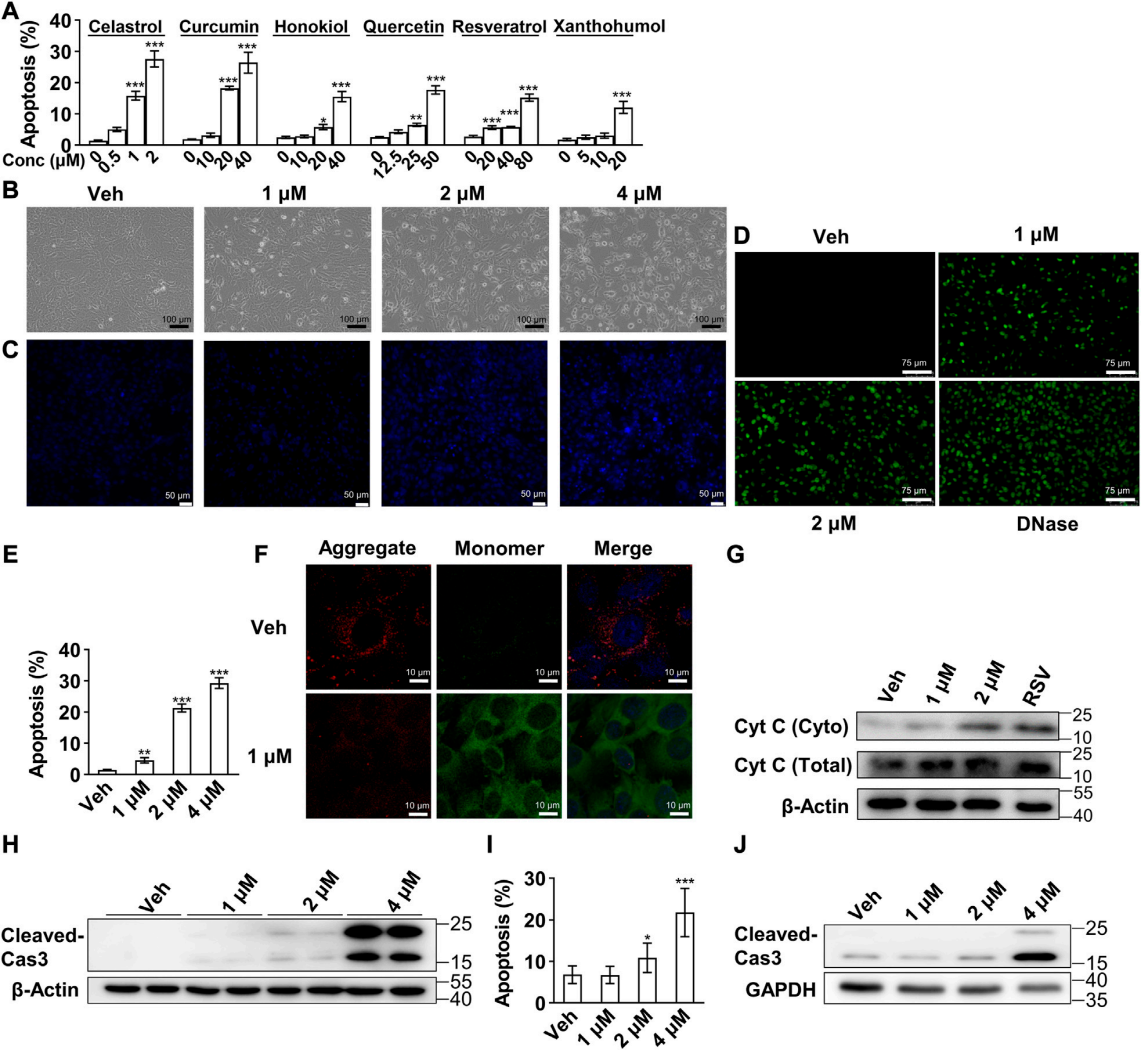
Celastrol induced 3T3-L1 preadipocytes apoptosis *via* mitochondrial mediated pathway **(A)**, Flow cytometry of apoptosis assay of 3T3-L1 preadipocytes after 24 h treatment of celastrol (0, 0.5, 1 and 2 μM), curcumin (0, 10, 20 and 40 μM), honokiol (0, 10, 20 and 40 μM), quercetin (0, 12.5, 25 and 50 μM), resveratrol (0, 20, 40 and 80 μM) or xanthohumol (0, 5, 10 and 20 μM) (*n* = 7–10) **(B–D)**, 3T3-L1 preadipocytes after 24 h treatment of 0, 1, 2 and 4 μM celastrol were subjected to light microscope imaging **(B)**, Hoechst 33258 staining **(C)**, Terminal deoxynucleotidyl transferase dUTP nick end labelling (TUNEL) staining **(D)** (*n* = 4) **(E)**, 3T3-L1 preadipocytes after 16 h treatment of 0, 1, 2 and 4 μM celastrol were subjected to flow cytometry analysis (*n* = 7) **(F)**, 3T3-L1 preadipocytes after 24 h treatment of 1 μM celastrol were subjected to JC-10 dye staining (*n* = 4) **(G)**, Western blotting of Cytochrome C (Cyt C) was conducted with cytoplasm extracts of preadipocytes after 1, 2 μM celastrol and 100 μM resveratrol treatment for 24 h (*n* = 3), resveratrol treatment was applied as a positive control **(H)**, Western blotting of cleaved-Caspase 3 was developed in preadipocytes after 1, 2 and 4 μM celastrol treatment for 24 h (*n* = 3) **(I–J)**, 3T3-L1 adipocytes were treated with 0, 1, 2 and 4 μM celastrol for 24 h, and subjected to flow cytometry analysis **(I)** (*n* = 6) and Western blotting of cleaved-Caspase3 **(J)** (*n* = 3). Error bars represent SEM; *p < 0.05; **p < 0.01; ***p < 0.001. Veh, vehicle; Cas 3, Caspase 3.

Induction of preadipocytes apoptosis represents a potential anti-obesity treatment strategy, and our previous study of resveratrol and other studies showed several phytochemicals with this effect (Hsu and Yen, 2006; Yang et al., 2007; Chen et al., 2012; Zhang and Huang, 2012; Lone and Yun, 2017; Wu et al., 2019). In consideration that celastrol was reported as one of the most robust anti-obesity phytochemicals (Liu et al., 2015), we compared the induced apoptosis effects of these compounds with celastrol. As shown in Figure 1A, a rather lower concentration of celastrol illustrated the strongest efficacy on preadipocytes apoptosis compared to other compounds after 24 h treatment. Furthermore, 3T3-L1 preadipocytes were subjected to 1, 2 and 4 μM celastrol for 24 h, and the pathomorphological alteration of apoptosis of preadipocytes as verified with light microscope (Figure 1B), condensed nuclei (Figure 1C) and fracture of genome DNA (Figure 1D) were observed. A concentration dependent effect of celastrol on preadipocytes apoptosis was also observed after 16 h treatment (Figure 1E).

To determine the mechanism underlying celastrol-induced apoptosis, we first observed enhanced green fluorescence after celastrol treatment with the JC-10 fluorescence probe, showing that mitochondria membrane potential was impaired after celastrol treatment (Figure 1F). Next, Western blotting illustrated a concentration dependent release of Cytochrome C in cytoplasm extracts (Figure 1G), and accumulation of cleaved-Caspase 3 in whole cell extracts after celastrol treatment (Figure 1H). These results indicated that celastrol activated the intrinsic apoptosis *via* mitochondrial mediated pathway in preadipocytes. Lastly, the effect of celastrol was also investigated in 3T3-L1 mature adipocytes. As shown in flow cytometry (Figure 1I) and Western blotting (Figure 1J), 24 h celastrol treatment exhibited similar apoptotic effects on mature adipocytes.

### 3.2 Celastrol induced 3T3-L1 preadipocytes apoptosis through inhibition of autophagy

**FIGURE 2:**
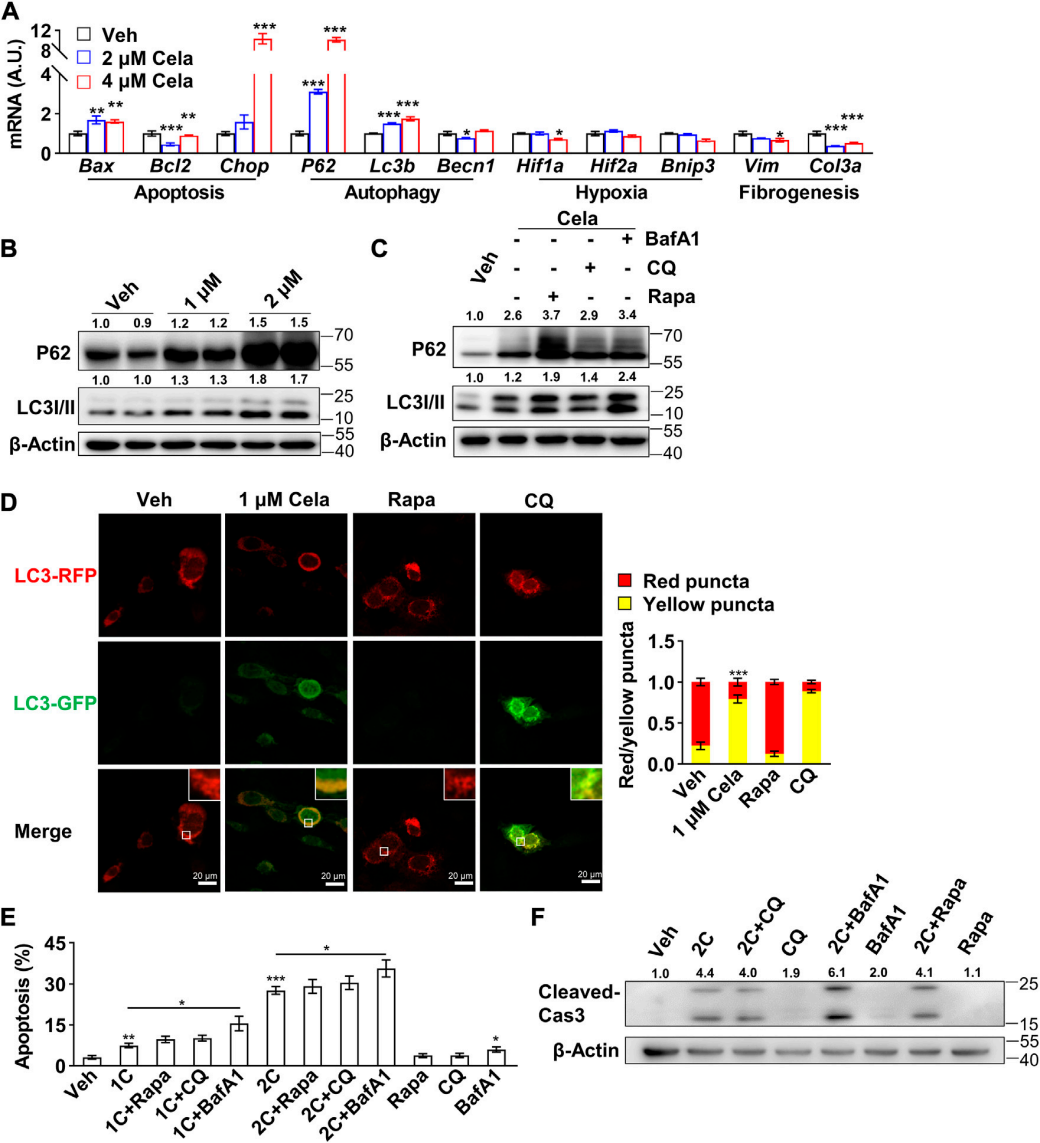
Celastrol induced 3T3-L1 preadipocytes apoptosis through inhibition of autophagy **(A)**, Real-time qPCR of genes were studied in 3T3-L1 preadipocytes after 0, 2 and 4 μM celastrol treatment for 16 h (*n* = 6) **(B)**, Western blotting of P62 and LC3 I/II were developed in 3T3-L1 preadipocytes after 0, 1 and 2 μM celastrol treatment for 24 h (*n* = 3) **(C)**, Western blotting of P62 and LC3 I/II were developed in preadipocytes after 2 μM celastrol treatment for 24 h with or without 15 μM rapamycin, 40 μM chloroquine or 200 nM bafilomycin A1 (*n* = 3) **(D)**, Autophagosome degradation was observed with RFP-GFP-LC3 adeno-associated virus, after 24 h treatment of 1 μM celastrol, 40 μM chloroquine or 15 μM rapamycin, respectively (*n* = 7). Red fluorescence represented normal autophagosome degradation, while yellow represented halt of degradation **(E–F)**, 3T3-L1 preadipocytes were treated with 24 h of celastrol, 15 μM rapamycin, 40 μM chloroquine, 200 nM bafilomycin A1, celastrol + 15 μM rapamycin, celastrol + 40 μM chloroquine or celastrol + 200 nM bafilomycin A1, respectively, and subjected to flow cytometry analysis **(E)** (*n* = 7) and Western blotting of cleaved-Caspase3 **(F)** (*n* = 3). Protein expression was calculated relative to *β*-actin and depicted at the top of each blot. Error bars represent SEM; *p < 0.05; **p < 0.01; ***p < 0.001. Veh, vehicle; Cela, celastrol; 1C, 1 μM celastrol; 2C, 2 μM celastrol; BafA1, bafilomycin A1; CQ, chloroquine; Rapa, rapamycin; Cas 3, Caspase 3.

In consideration that autophagy (Gordy and He, 2012), hypoxia (Sendoel and Hengartner, 2014) and fibrogenesis (Mehal and Imaeda, 2010) pathways were reported intensively interplay with apoptosis pathway to maintain cell viability, we hypothesized that celastrol might induce apoptosis of preadipocytes through regulation of these pathways. We first applied Real-time qPCR to search the potential pathway. As shown in Figure 2A, after celastrol treatment, the pro-apoptotic genes: *Bax* and *Chop* were significantly upregulated and the anti-apoptotic gene *Bcl2* was significantly downregulated. The autophagy-related genes: *P62* and *Lc3b* showed significantly upregulation, while *Becn1* showed a mere alteration. However, no significant changes were observed in the hypoxia-related genes: *Hif1a*, *Hif2a* and *Bnip3*. The fibrogenesis-related genes: *Vim* and *Col3a* showed downregulation. Therefore, we believed celastrol induced apoptosis of preadipocytes through regulation of autophagy. Furthermore, the inhibition of autophagic flux was also verified with accumulation of P62 and LC3 II after celastrol treatment, using Western blotting (Figure 2B). Autophagy involves three main steps: 1) the formation of double membrane-bound vesicles called autophagosomes, 2) the fusion of autophagosomes and lysosomes, and 3) the acidification of autophagolysosomes (Mauvezin et al., 2015). The abrogation of each step would lead to the halt of the whole autophagic flux. To further verify the exact autophagy step in which celastrol regulates, rapamycin, an activator of autophagy initiation and autophagosome formation, was firstly applied in addition to celastrol. If celastrol inhibits the formation of autophagosome, it is plausible that rapamycin can block the inhibitory effect of celastrol on autophagy. Conversely, we observed increased accumulation of P62 and LC3 II after this treatment, indicating that celastrol probably targets the downstream of autophagic flux (Figure 2C). Furthermore, chloroquine, an inhibitor only targeting step 2 (the fusion of autophagosomes and lysosomes) (Mauthe et al., 2018), and bafilomycin A1, an inhibitor targeting step 2 and step 3 (the acidification of autophagolysosomes) (Mauvezin et al., 2015), were applied in addition to celastrol. We observed that only celastrol + bafilomycin A1 group, but not celastrol + chloroquine group, showed increased accumulation of P62 and LC3II comparing to the celastrol group (Figure 2C). Therefore, we deduced that celastrol might have a similar effect with the chloroquine and only inhibit the fusion of autophagosomes and lysosomes.

To further verify celastrol’s effect on autophagy, RFP-GFP-LC3 adeno-associated virus was applied to monitor autophagosome degradation. The number of autophagic vacuoles and vesicles containing RFP-GFP-LC3 was markedly increased after celastrol treatment, indicating the impairment of autophagosome degradation. Similar phenomenon was observed after chloroquine treatment, while rapamycin treatment exhibited normal red fluorescence (Figure 2D). Last, the combination effects of celastrol and these three compounds on apoptosis were studied. As observed by flow cytometry (Figure 2E) and cleavage of Caspase3 (Figure 2F), celastrol + chloroquine group and celastrol + rapamycin group exhibited comparable apoptotic effect again celastrol group, whereas celastrol + bafilomycin A1 group showed higher apoptotic effect. We speculated that celastrol might share similar mechanism with chloroquine, targeting the inhibition of autophagosomes and lysosomes fusion, since their combination did not show superimposed effect.

### 3.3 Celastrol inhibited the fusion of autophagosome and lysosome

**FIGURE 3:**
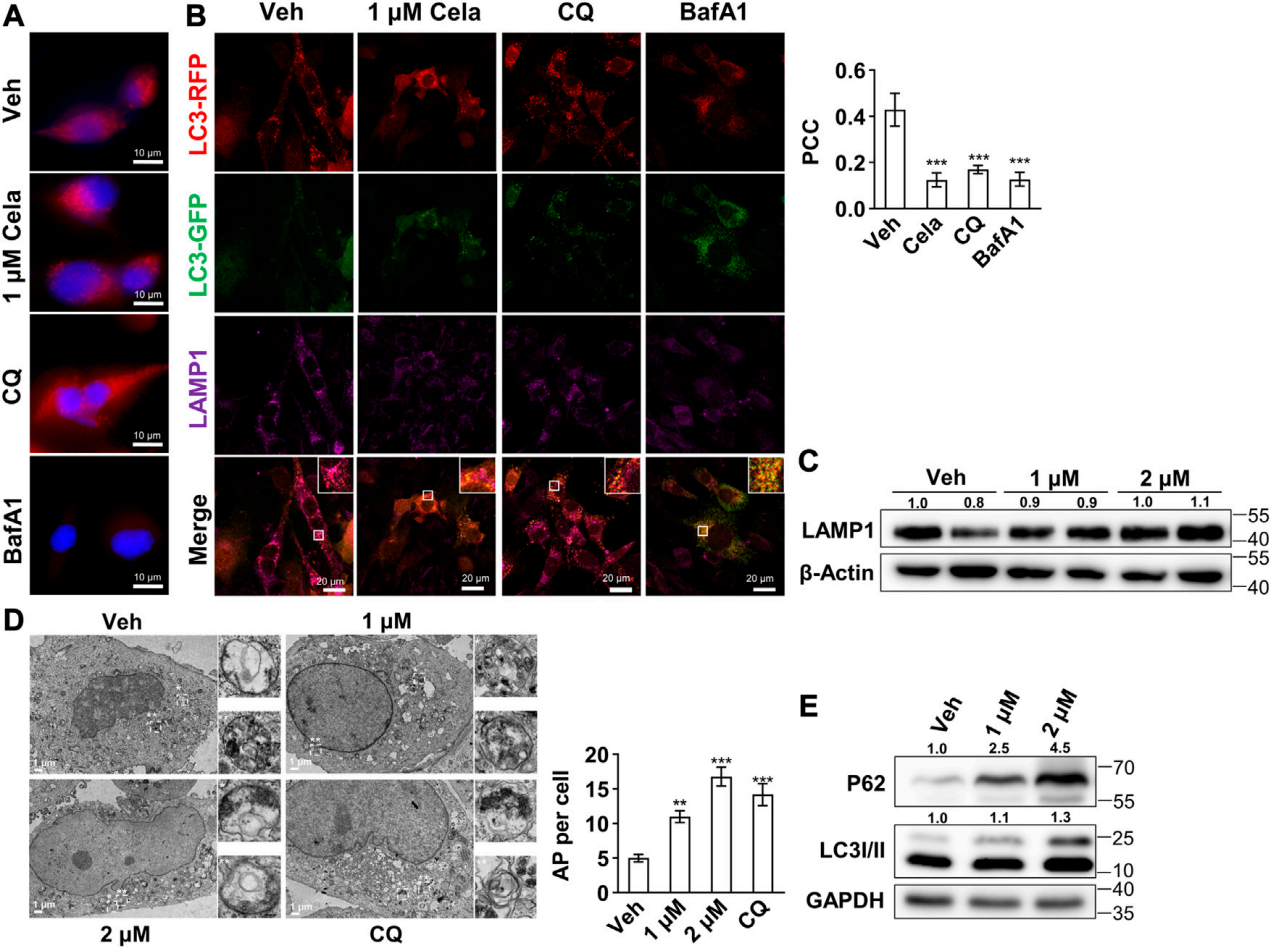
Celastrol inhibited the fusion of autophagosome and lysosome **(A–B)**, 3T3-L1 preadipocytes were treated with 24 h of 1 μM celastrol, 40 μM chloroquine or 200 nM bafilomycin A1, and subjected to LysoTracker red staining **(A)** (*n* = 3) and immunofluorescence staining of LAMP1 and LC3 **(B)**. The representative images were shown on the left, and the Pearson correlation coefficient (PCC) for the colocalization of RFP-LC3 and Alexa Fluor 647-LAMP1 were presented on the upper right (*n* = 3) **(C)**, 3T3-L1 preadipocytes were treated with 1 and 2 μM celastrol for 24 h and subjected to Western blotting of LAMP1 (*n* = 3) **(D)**, 3T3-L1 preadipocytes were treated with 1, 2 μM celastrol and 40 μM chloroquine for 12 h and subjected to electron microscopy (*n* = 5) **(E)**, 3T3-L1 adipocytes were treated with 1 and 2 μM celastrol for 24 h and subjected to Western blotting of P62 and LC3 I/II (*n* = 3). Protein expression was calculated relative to *β*-actin or GAPDH and depicted at the top of each blot. Error bars represent SEM. Veh, vehicle; Cela, celastrol; CQ, chloroquine; BafA1, bafilomycin A1; AP, autophagosome.

To further validate our hypothesis that celastrol mainly inhibits the autophagosome and lysosome fusion, we first tested the lysosome acidification after intervention. As shown in Figure 3A, no decrease of red puncta was observed in celastrol and chloroquine group, indicating no effect on lysosome acidification. Bafilomycin A1, however, exhibited significant decrease of red puncta, indicating an abrogation of lysosome acidification. Furthermore, the colocalization of autophagosome marked with RFP-GFP-LC3 and lysosome marked with its membrane protein LAMP1 was studied. Decreased colocalization was observed in celastrol, chloroquine and bafilomycin A1 groups (Figure 3B), whereas LAMP1 protein was not altered after celastrol treatment (Figure 3C). The accumulation of autophagosomes after celastrol and chloroquine treatment was also directly observed *via* electron microscopy (Figure 3D). These data indicated the inhibition of autophagosome and lysosome fusion after celastrol treatment. Considering the vital role of autophagy in adipogenesis (Baerga et al., 2009; Zhang et al., 2009), we also observed a significant inhibition of differentiation of human primary visceral preadipocytes with low doses of celastrol (200 nM–800 nM) (Supplementary Figure S1). Moreover, the inhibition of autophagic flux upon celastrol treatment was also confirmed on 3T3-L1 mature adipocytes as determined by the Western blotting of P62 and LC3 II (Figure 3E).

### 3.4 Celastrol bond with VAMP7 and RAB7 to inhibit autophagy and subsequently induce apoptosis

**FIGURE 4:**
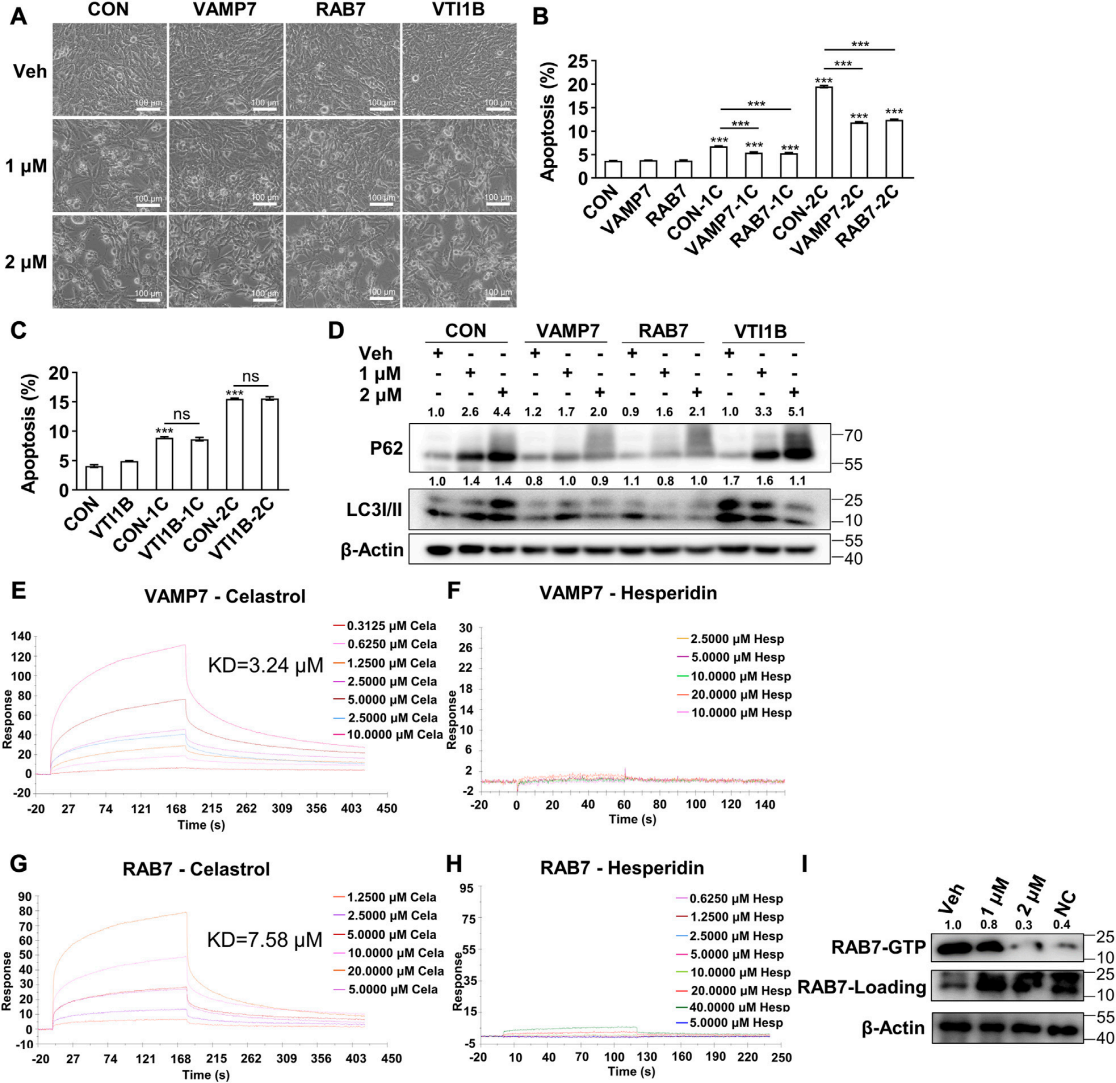
Celastrol bond with Vamp7 and Rab7 to inhibit autophagy and subsequently induce apoptosis **(A–D)**, 3T3-L1 preadipocytes were transfected with blank, Vamp7, Rab7 and Vti1b plasmids for 48 h, and then treated with 0, 1 and 2 μM celastrol for 24 h and subjected to phase contrast light microscope imaging **(A)** (*n* = 4), flow cytometry analysis **(B, C)** (*n* = 4), and Western blotting of P62 and LC3 I/II **(D)** (*n* = 4) **(E–H)**, Surface Plasmon Resonance studies of VAMP7 with celastrol **(E)** and hesperidin **(F)**, RAB7 with celastrol **(G)** and hesperidin **(H)** were shown. **(I)** RAB7-GTP pull-down assay was performed in preadipocytes after 1 and 2 μM celastrol treatment for 16 h and shown by Western blotting of RAB7-GTP and total RAB7 (*n* = 3). Protein expression was calculated relative to *β*-actin and depicted at the top of each blot. Error bars represent SEM; ns, no significance; ***p < 0.001. Veh, vehicle; CON, control; Cela, C, celastrol; 1C, 1 μM celastrol; 2C, 2 μM celastrol; NC, negative control.

In light of the abovementioned results, we hypothesized that celastrol might directly bind with certain proteins during the fusion of autophagosome and lysosome, and subsequently inhibit the autophagic flux. To determine the direct binding protein of celastrol, the components mediating autophagosome and lysosome fusion were applied for virtual docking with celastrol (Supplementary Figure S2). Notably, VAMP7, RAB5C, RAB7 and VTI1B were the top four candidate proteins with the strongest binding potential with celastrol. Therefore, the genic manipulation studies of VAMP7, RAB5C, RAB7 and VTI1B were applied. Importantly, overexpression of VAMP7 or RAB7 significantly inhibited celastrol induced apoptosis in 3T3-L1 preadipocytes (Figures 4A,B, Supplementary Figure S2), whereas no effect was observed with overexpression of VTI1B (Figures 4A, C, Supplementary Figure S2) or knockdown of RAB5C (Supplementary Figure S2) in the combination with celastrol. Next, these manipulations on celastrol induced autophagic flux inhibition was also investigated. As shown in Figure 4D, overexpression of VAMP7 or RAB7 significantly reversed the accumulation of P62 and LC3II after celastrol treatment in 3T3-L1 preadipocytes, while overexpression of VTI1B showed no effect. Likewise, knockdown of RAB5C exhibited no effect on autophagy after celastrol treatment (Supplementary Figure S2). Taken together, these data suggested that VAMP7 and RAB7, not VTI1B or RAB5C, intervened the effect of celastrol on autophagy and apoptosis, which might be the direct target of celastrol.

To further verify the direct binding target, we purified VAMP7 and RAB7 (Supplementary Figure S2) and performed Surface Plasmon Resonance study. The results showed a direct binding of celastrol with VAMP7 (Kd = 3.24 μM, Figure 4E), as well as celastrol with RAB7 (Kd = 7.58 μM, Figure 4G). Meanwhile, hesperidin, another phytochemical, showed no binding with VAMP7 (Figure 4F) or RAB7 (Figure 4H). We further studied RAB7 GTPase activity with a RAB7-GTP pull-down assay. As the initial switch of these interactions and the following membrane fusion, RAB7 GTPase activity was significantly reduced after celastrol treatment, as indicated by the decrease of RAB7-GTP against total RAB7 (Figure 4I). These data confirmed that VAMP7 and RAB7 were the direct binding targets of celastrol, and celastrol inhibited the GTPase activity of RAB7 *via* direct binding.

### 3.5 Celastrol induced apoptosis and inhibited autophagy in murine epididymal adipose tissue and human primary visceral preadipocytes.

**FIGURE 5:**
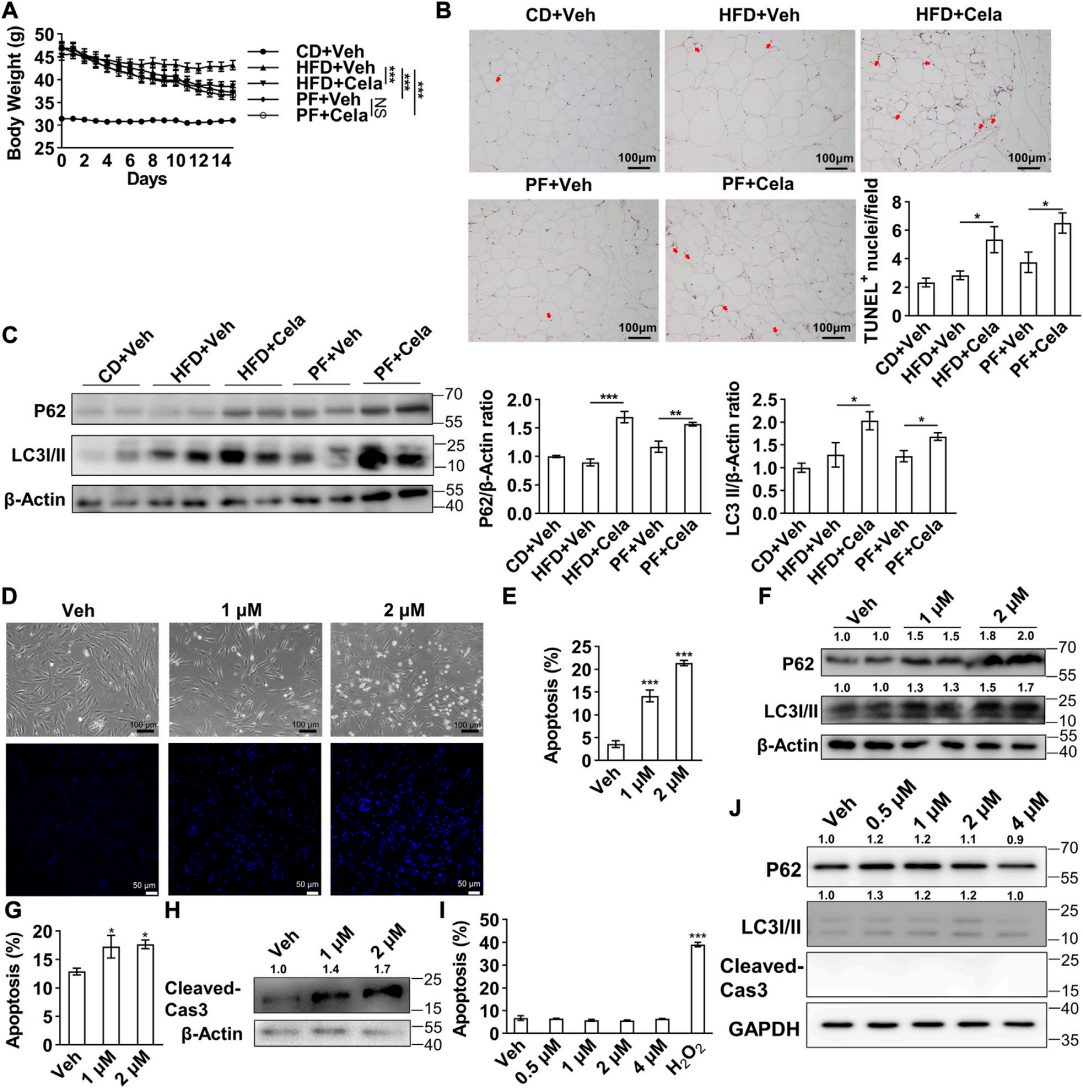
Celastrol induced apoptosis and inhibited autophagy in murine epididymal adipose tissue and human primary visceral preadipocytes **(A–C)**, 4-week-old male C57BL/6 mice were fed with 60% high fat diet for 24 weeks to induce diet-induced obesity. Mice were randomly divided into five groups, 1) chow diet + vehicle, 2) high fat diet + vehicle, 3) high fat diet + celastrol, 4) pair-feeding + vehicle and 5) pair-feeding + celastrol (n = 4). For 2 weeks intervention of celastrol, mice in pair-feeding group only received equal amount diet comparing with celastrol treatment group to mimic the anorexia effect of celastrol. Body weight change were shown in **(A)**. The murine epididymal adipose tissue was further harvested and subjected to TUNEL staining **(B)**, and Western blotting of P62 and LC3 I/II **(C)**, the representative images were shown on the left, quantification of P62 and LC3 I/II were shown on the right) **(D–F)**, Human primary visceral preadipocytes were treated with 0, 1 and 2 μM celastrol and subjected to light microscope imaging and Hoechst 33258 staining **(D)**, flow cytometry analysis **(E)** (*n* = 4) and Western blotting of P62 and LC3 I/II **(F)** (*n* = 3) **(G–H)**, Human primary visceral adipocytes were treated with 0, 1 and 2 μM celastrol for 24 h and subjected to flow cytometry analysis **(G)** (*n* = 6) and Western blotting of cleaved-Caspase3 **(H)** (*n* = 3) **(I–J)**, Human hepatocytes HL-7702 were treated with 0, 1, 2 and 4 μM celastrol for 24 h and subjected to flow cytometry analysis **(I)** (*n* = 6) and Western blotting of P62, LC3 I/II and cleaved-Caspase3 **(J)** (*n* = 3). Protein expression was calculated relative to *β*-actin or GAPDH and depicted at the top of each blot. Error bars represent SEM; *p < 0.05; ***p < 0.001. Veh, vehicle; Cela, celastrol; Cas 3, Caspase 3; CD + Veh, chow diet + vehicle; HFD + Veh, high fat diet + vehicle; HFD + Cela, high fat diet + celastrol; PF + Veh, pair-feeding + vehicle; PF + Cela, pair-feeding + celastrol.

To validate celastrol’s effect in murine fat tissue, we further performed *in vivo* study using diet-induced obese mice treated with celastrol. Given that Liu et al. firstly reported up to 79% food intake reduction after celastrol administration in mice (Liu et al., 2015), we applied the pair-feeding group given equal amount diet (about 21%) per day, as a control to exclude the potential side-effects due to food reduction. Diet-induced obese mice were randomly divided into five groups including 1) chow diet + vehicle (CD + Veh), 2) high fat diet + vehicle (HFD + Veh), 3) high fat diet + celastrol (HFD + Cela), 4) pair-feeding + vehicle (PF + Veh) and 5) pair-feeding + celastrol (PF + Cela). After 2 weeks intervention of celastrol, significant decrease of body weight was observed in HFD + Cela group *versus* HFD + Veh group, whereas pair feeding groups showed similar decrease of body weight comparing with HFD + Cela group. No significant body weight change was observed in PF + Veh group and PF + Cela group (Figure 5A). We further harvested murine epididymal adipose tissue from these five groups for apoptosis and autophagy study. The TUNEL staining of epididymal adipose tissue showed a slight increase of apoptosis in celastrol treatment groups comparing with the corresponding vehicle groups (Figure 5B). Using Western blotting of P62 and LC3 I/II, we found that significant accumulation of both P62 and LC3 II in celastrol treatment groups comparing with the corresponding vehicle groups (Figure 5C). These *in vivo* findings were in consistence with our *in vitro* results, showing direct inhibitory effect on autophagy and pro-apoptosis effect of celastrol on visceral fat tissue, apart from its anorexia effect.

The induced apoptosis of celastrol might present a potential treatment strategy for obesity, however, efficacy of this compound in human remained unknown. Therefore, a direct induction of apoptosis of celastrol on human primary preadipocytes was observed as determined by morphological alteration of cells (Figure 5D). Furthermore, flow cytometry exhibited a dose-dependent apoptotic effect of celastrol (Figure 5E). Additionally, the accumulation of P62 and LC3 II was observed after celastrol treatment, confirming an inhibition of autophagic flux (Figure 5F). The induced apoptosis of celastrol was observed in human primary visceral mature adipocytes after 24 h treatment, as indicated by the flow cytometry (Figure 5G) and Western blotting of cleaved-Caspase3 (Figure 5H). Finally, to verify its potential safety in human, we applied the same dosage of celastrol on human hepatocytes HL-7702 for 24 h. No induction of apoptosis (Figure 5I) and inhibition of autophagy were observed (Figure 5J), suggesting a potential selectivity of celastrol on regulation of apoptosis and autophagy in human primary cells.

## 4 Discussion

Celastrol was reported as one of the most robust anti-obesity phytochemicals, yet its direct target remained unclear. In this study, we identified VAMP7 and RAB7 as the direct binding targets of celastrol, which mediate the regulatory effects of celastrol on cellular apoptosis and autophagy in preadipocytes. These findings clarified the direct effect of celastrol on preadipocytes and its underlying mechanism, which would broaden our understanding of the anti-obesity effect of celastrol.

In our study, celastrol demonstrated an effect of inhibiting the fusion of autophagosomes and lysosomes in preadipocytes, and we further found that VAMP7 and RAB7 were the direct targets of celastrol mediating its regulation on autophagy. During autophagosomes and lysosomes fusion, the soluble N-ethylmaleimide-sensitive fusion protein-attachment protein receptor (SNARE) complexes and Rab-GTPases participated in the trafficking between autophagosome and lysosomes (Dingjan et al., 2018; Langemeyer et al., 2018). VAMP7 was reported as one of the key components of the SNARE complex. Previous studies illustrated that overexpression of VAMP7 could increase autophagolysosomes (Fader et al., 2009). Meanwhile, RAB7 is a key member of Rab-GTPases and is required for autophagic pathway. For the initiation of autophagosome and lysosome fusion and degradation, RAB5C is the main endosomal GTPase, which is replaced by RAB7 during maturation of endosomes and lysosomes (Langemeyer et al., 2018). Studies in yeast showed that the RAB7-like Ypt7p mediated the anchoring of HOPS to the membrane (Hickey et al., 2009), which subsequently recruited and retained the VAMP7-like Vam7p (Ungermann et al., 2000). In light of these studies, our study showed overexpression of VAMP7 or RAB7 could reverse the inhibitory effect of celastrol on autophagy, which subsequently block the apoptotic effect. The Surface Plasmon Resonance study further confirmed the direct binding of celastrol with VAMP7 and RAB7. Further functional study illustrated the inhibition of RAB7 GTPase activity after celastrol treatment. Taken together, we proposed that celastrol directly bond with VAMP7 and RAB7 to inhibit autophagy.

Despite that autophagy was first observed under starvation and nutrients depleted status, recent studies illustrated an important role of autophagy in regulation of obesity. The crosstalk between autophagy and apoptosis is vital for cell hemostasis (Gordy and He, 2012). Cells utilize autophagy for recycling essential metabolites, such as lipids and amino acids for fueling the bioenergetic machinery (Doherty and Baehrecke, 2018). Therefore, when autophagy was blocked, apoptosis was induced with mitochondrial outer membrane permeabilization and subsequent a serial of caspases activation (Boya et al., 2005; González-Polo et al., 2005). Moreover, the inhibition of the fusion of autophagosomes and lysosomes could result in accumulation of autophagosomes, which would further sequestrate the essential nutrients required for metabolism. The data in our study revealed that celastrol inhibited the fusion of autophagosomes and lysosomes, and subsequently induced apoptosis *via* mitochondrial mediated pathway in preadipocytes. Moreover, the autophagy was documented closely connected with preadipocyte differentiation process. The inhibition of autophagy, by knockout of autophagy-related gene 5 (atg5) and atg7, would inhibit the adipogenesis both *in vitro* and *in vivo* (Baerga et al., 2009; Zhang et al., 2009). In line with these findings, we also observed a significant inhibition of preadipocyte differentiation and decrease of lipid accumulation after low concentration celastrol treatment, potentially due to the inhibition of autophagy. In consistence with our *in vitro* study, we observed comparable pro-apoptotic effect of celastrol in both murine epididymal adipose tissue and human mature adipocytes. Apart from our study, we must point out that excessive induction of preadipocytes apoptosis might abrogate the homeostasis of adipocyte metabolism, the appropriate dosage of celastrol in clinical translation should be further studied.

In conclusion, celastrol inhibits the fusion of autophagosomes and lysosomes *via* a direct binding with VAMP7 and RAB7, leading to accumulation of autophagosomes. Abrogation of autophagy by celastrol further induced apoptosis in preadipocytes and adipocytes, thus reducing excessively fat mass accumulation. These effects suggest a potential strategy of using celastrol for treating obesity.

## Data Availability

The raw data supporting the conclusions of this article will be made available by the authors, without undue reservation.
