# Supplementary material for: Celastrol directly binds with VAMP7 and RAB7 to inhibit autophagy and induce apoptosis in preadipocytes
Source: Front Pharmacol. 2023 Mar 7;14:1094584. doi: 10.3389/fphar.2023.1094584 (PMC10027750; doi:10.3389/fphar.2023.1094584)
Supplement: Supplementary file 1 [file DataSheet1.PDF]

Supplementary Material

1 Supplementary Figures

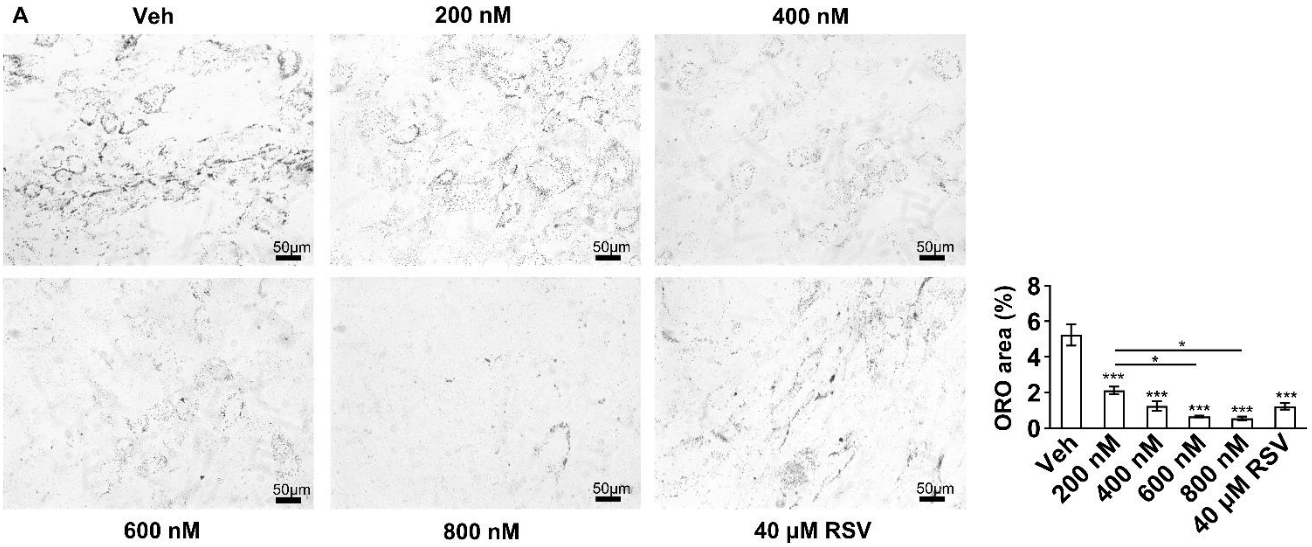

Supplementary Figure 1. Celastrol inhibited the differentiation of human primary visceral preadipocytes. (A), Human primary visceral preadipocytes were treated with 200 nM - 800 nM celastrol and 40 µM resveratrol during the differentiation and subjected to Oil Red O staining (n = 6), resveratrol was applied as positive control. Error bars represent SEM; ***p < 0.001. Veh, vehicle.

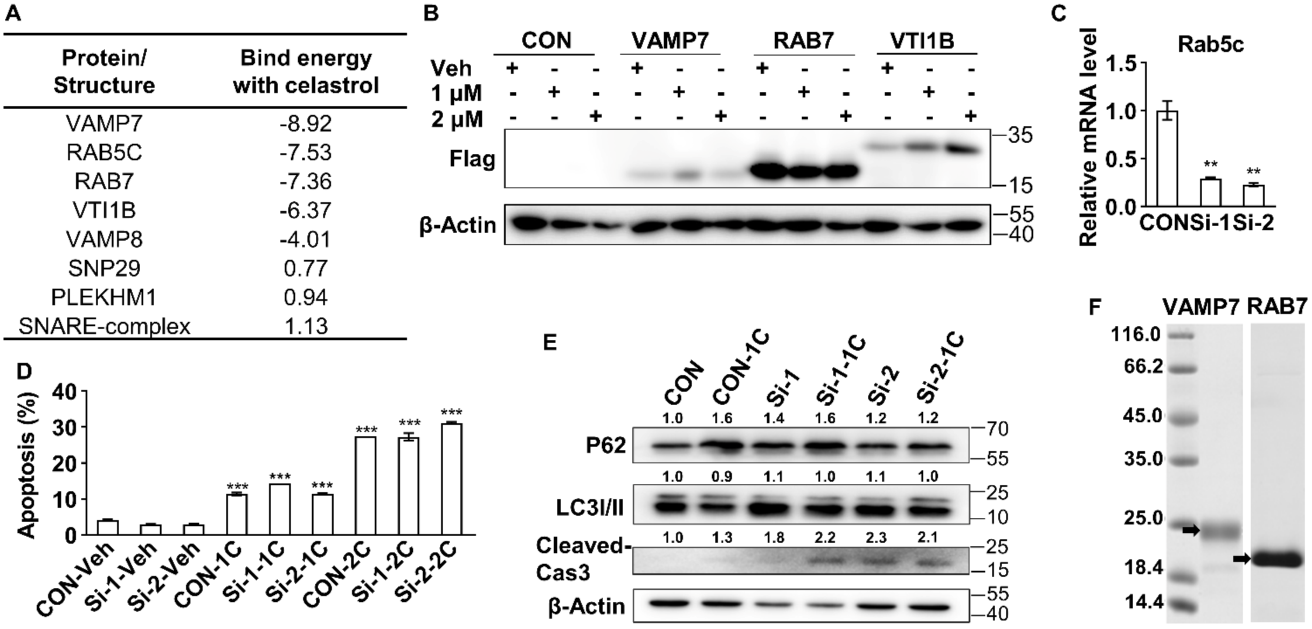

Supplementary Figure 2. Celastrol bond with Vamp7 and Rab7 to inhibit autophagy and subsequently induce apoptosis. (A), Binding energy of proteins with celastrol was shown. **(B)**, 3T3-L1 preadipocytes were transfected with blank, Rab7, Vamp7 or Vti1b plasmids for 48 h, and then treated with 0, 1 and 2 μM celastrol for 24 h and subjected to western blotting of Flag (n = 4). **(C)**, qPCR of Rab5c was applied in verification of knockdown efficacy of siRNA of Rab5c (n = 3). **(D-E)**, 3T3-L1 preadipocytes were transfected with 10 nM scramble siRNA and Rab5c siRNA for 24 h, and then treated with 0, 1 and 2 μM celastrol for 24 h and subjected to flow cytometry analysis **(D)** (n = 3) and western blotting of cleaved caspase 3, P62 and LC3 I/II **(E)** (n = 3). **(F)**, Purified VAMP7 and RAB7 were determined by Coomassie brilliant blue staining (arrow indicated). Protein expression was calculated relative to β-actin and depicted at the top of each blot. Error bars represent SEM; $^{**}p < 0.01$; $^{***}p < 0.001$. CON, control; 1C, 1 μM celastrol; 2C, 2 μM celastrol; SNARE; Soluble N-ethylmaleimide-sensitive fusion protein-attachment protein receptor; Cas3, Caspase3.
